# When calculation of minimum sample size is not justified: When sample size calculation is not justified

**Published:** 2011-03-01

**Authors:** Mohamad Amin Pourhoseingholi, Ahmad Reza Baghestani

**Affiliations:** 1 Research Center for Gastroenterology and Liver Diseases, Shahid Beheshti University of Medical Sciences, Tehran, IR Iran; 2 Department of Mathematics, Islamic Azad University, South Tehran Branch, Tehran, IR Iran

**Keywords:** Sample size, Clinical trial

Dear Editor,

We read with interest the paper of Estévez, et al, recently published in Hepatitis Monthly [1]. That article was a Letter to the Editor on the study conducted by Nikui Nejad et al [2]. One of the comments made by Estévez et al was on the adequacy of the size of the studied sample. They believed that the sample size was not adequate based on the provided data and the type of study [1]. In the reply from the authors, a familiar mathematical equation which is frequently used for prevalence study designs was presented to explain the way the sample size was calculated in Nikui Nejad's study [1].

In spite of doubts in the statistical analyses used in their study, we should emphasize that the study of Nikui Nejad, et al, was clearly a clinical trial involving comparison between two vaccines. In that study, the sample size did not depend on the prevalence of vaccine response rate in the population-instead, it did depend on the difference between the response rates from the two vaccines. The authors also used an incorrect assumption for the calculation of minimum sample size according the "prevalence approach" and assumed a large d with a small P! We know that in clinical trial, if the sample size is too small, a well-conducted study may fail to answer its research hypothesis or to detect important effects and associations. Therefore, correct assumptions for the calculation of minimum sample size are of paramount importance in conducting research. Approaches for estimating sample size and performing power analysis depend primarily on the study design and the main outcome measure [3]. Clinical trials should be large enough to detect reliably the smallest possible differences in the primary outcomes. It is not uncommon for studies to be underpowered, failing to detect even large treatment effects because of inadequate sample size [4]. The minimum information needed to calculate sample size for a randomized controlled trial includes the study power (1-ß), the level of significance (α), the underlying event rate in the population and the effect size. The calculated minimum sample size should then be adjusted for other factors, including expected compliance rates and, less commonly, an unequal allocation ratio. The objectives and outcome measures of the study must be clearly stated, and the information used in calculating the minimum sample size should reflect as closely as possible the type of data that will be gathered from the proposed trial [5]. Sample size calculation is an important part of any clinical trials and a professional statistician is the best person to be asked for help at the time of planning a research project. However, researchers must be prepared to provide the necessary information so that the sample size can be determined [6]. There are many statistical books on the methods for sample size calculation in medical studies [7]. There are also several software programs available to help with sample size calculations. While these programs are easy to use, investigators should consult biostatisticians at the design stages of their projects and any article containing even the most elementary statistical procedure should be reviewed by an expert biostatistician.
